# Lipodystrophy in HIV: Evolving Challenges and Unresolved Questions

**DOI:** 10.3390/ijms26146546

**Published:** 2025-07-08

**Authors:** Marta Giralt, Pere Domingo, Tania Quesada-López, Rubén Cereijo, Francesc Villarroya

**Affiliations:** 1 Department of Biochemistry and Molecular Biomedicine, Institute of Biomedicine of the University of Barcelona (IBUB), 08028 Barcelona, Spain; tquesada@ub.edu (T.Q.-L.); rcereijo@ub.edu (R.C.); 2 CIBER “Fisiopatología de la Obesidad y Nutrición” (CIBEROBN), Instituto de Salud Carlos III, 28029 Madrid, Spain; 3 Infectious Diseases Unit, Hospital de la Santa Creu i Sant Pau, 08028 Barcelona, Spain; pdomingo@santpau.cat

**Keywords:** lipodystrophy, HIV, antiretroviral treatment, lipoatrophy, obesity, lipomatosis

## Abstract

The advent of effective antiretroviral therapy in the mid-1990s, which successfully prevented the progression to AIDS in people living with HIV (PLWH), was associated with the appearance of the so-called HIV-associated lipodystrophy. This condition involved subcutaneous fat atrophy; abdominal fat hypertrophy; and, in some cases, lipomatosis. It was also associated with systemic metabolic disturbances, primarily insulin resistance and dyslipidemia. Following the replacement of certain antiretroviral drugs, particularly the thymidine-analog reverse transcriptase inhibitors stavudine and zidovudine, with less toxic alternatives, the incidences of lipoatrophy and lipomatosis significantly declined. However, lipodystrophy resulting from first-generation antiretroviral therapy does not always resolve after switching to newer agents. Although the widespread use of modern antiretroviral drugs—especially integrase strand transfer inhibitors and non-lipoatrophic reverse transcriptase inhibitors such as tenofovir alafenamide—has reduced the incidences of severe forms of lipodystrophy, these regimens are not entirely free of adipose tissue-related effects. Notably, they are associated with weight gain that resembles common obesity and can have adverse cardiometabolic consequences. Recent evidence also suggests the hypertrophy of specific fat depots, such as epicardial and perivascular adipose tissue, in PLWH on last-generation treatments, potentially contributing to increased cardiovascular risk. This evolving landscape underscores the persistent vulnerability of PLWH to adipose tissue alterations. While these morphological changes may not be as pronounced as those seen in classic HIV-associated lipodystrophy, they can still pose significant health risks. The continued optimization of treatment regimens and the vigilant monitoring of adipose tissue alterations and metabolic status remain essential strategies to improve the health of PLWH.

## 1. Introduction

The development of effective treatments for human immunodeficiency virus (HIV) infection has been one of the most significant therapeutic successes of recent decades. The expansion of the HIV epidemic in the 1980s led to high mortality rates associated with the immunodeficiency caused by the virus, known as acquired immunodeficiency syndrome (AIDS). This could only be mitigated after the mid-1990s through the development of combined antiretroviral drugs that effectively prevented the progression to AIDS and transformed the disease into a chronic condition. However, it was soon observed that, despite the success in preventing AIDS in treated patients, a complex syndrome involving alterations in the amount and distribution of adipose tissue developed, which came to be called HIV-associated lipodystrophy.

**Figure 1:**
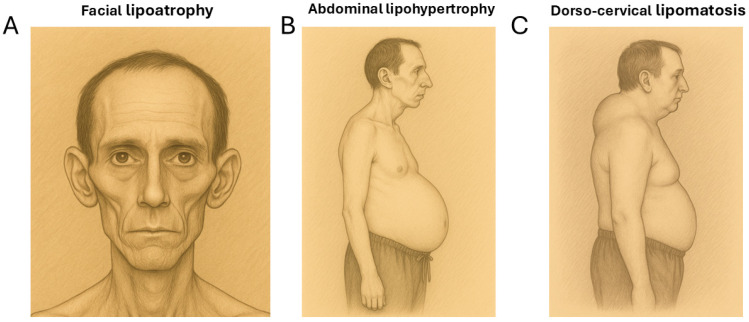
The pictorial representations of the main features of HIV-associated lipodystrophy. (**A**) Facial lipoatrophy. (**B**) Abdominal lipohypertrophy. (**C**) A lipomatous “buffalo hump” adipose tissue accumulation in the dorso-cervical region.

The lipodystrophy observed in people living with HIV (PLWH) is a partial type that has complex manifestations. Firstly, there is subcutaneous fat loss (lipoatrophy) in the face, arms, and legs. Secondly, there is also fat hypertrophy in the abdominal area, the reminiscent of visceral obesity. Lastly, less frequently than the previous phenomena, lipomatous accumulations appear in the dorso-cervical area (commonly called “buffalo humps”) and sometimes appear in other regions. All these changes can occur simultaneously in the same individual, or, at times, patients may predominantly manifest only one type of alteration [1,2,3] (Figure 1).

Morphological changes in the amount and distribution of fat in PLWH are associated with systemic metabolic alterations partly resembling those observed in the genetic forms of partial lipodystrophy. These include insulin resistance, type 2 diabetes, dyslipidemia, hepatic steatosis, and elevated cardiovascular risk [3,4]. In fact, these metabolic disturbances are among the most significant health issues linked to HIV-associated lipodystrophy. However, other factors—such as the stigmatizing effects of facial lipoatrophy—also greatly affect the quality of life of PLWH [5].

HIV-associated lipodystrophy showed a remarkable prevalence until the early 2020s, although the available data on the global prevalence of lipodystrophy (including lipoatrophy and/or lipohypertrophy) vary widely. In studies based on the clinical diagnoses of lipodystrophy, the reported prevalence of HIV-associated lipodystrophy in PLWH receiving combined antiretroviral therapy (cART) that included first-generation drugs ranged from 23% to 70%. This wide variation in prevalence was attributed to differences in study design and the length of follow-up across studies [6]. Subsequently, in the 2020s, thanks to the development of highly effective antiretroviral drugs with minimal toxicity and treatments starting in the early stages post-infection, the occurrence of novel cases of overt lipodystrophy in PLWH has decreased dramatically. However, the persistence of relevant adipose tissue alterations that are not as obvious as overt lipoatrophy or lipomatosis in PLWH and the continued presence of lipodystrophy in PLWH who have been on treatment for many years (see later) remain significant challenges for maintaining a healthy status in PLWH.

## 2. Causal Agents of Lipodystrophy in PLWH

The causal agents of lipodystrophy in PLWH are multiple and complex, involving both the effects of antiretroviral treatments and the consequences of the underlying HIV infection. Additionally, it appears that the different manifestations of lipodystrophy (lipoatrophy, lipohypertrophy, and lipomatosis) may have distinct causal factors during their development.

### 2.1. Lipoatrophy

Regarding lipoatrophy, it historically manifested from the use of the initial antiretroviral therapy, which was based on a combination of three drugs, a protease inhibitor (PI) and two nucleoside reverse transcriptase inhibitors (NRTIs), which were, in most cases, thymidine analogs such as stavudine or zidovudine. It was soon observed that the successful maintenance of CD4 T cell levels in people with HIV was accompanied by marked subcutaneous fat loss, which in many cases could involve losing up to 30% of the fat in the extremities and a noticeable facial fat reduction [7]. It was noted that the presence of thymidine-analog NRTIs (stavudine and zidovudine) during treatment was preferentially associated with the appearance of lipoatrophy [8]. This was later corroborated after the dramatic reduction in lipoatrophy seen when thymidine-analog NRTIs were replaced with other antiretroviral drugs that were developed more recently and have lower toxicity [9]. By the late 2000s, it was recommended to avoid using zidovudine as an NRTI during treatment to prevent lipoatrophy, and the use of tenofovir difumarate (TDF), a newer nucleotide analog inhibiting HIV reverse transcriptase, became widespread. This drug has less lipoatrophic action (but not none) [10,11]. More recently, TDF has been replaced by tenofovir alafenamide (TAF), an analog of TDF devoid of lipoatrophic effects [12]. Additionally, some other antiretroviral drugs previously used to treat PLWH, such as efavirenz, a non-nucleoside inhibitor of reverse transcriptase (NNRTI), have also been shown to exert lipoatrophic actions [12]. There are currently significantly fewer cases of lipoatrophy in PLWH under the current patterns of antiretroviral treatments, although individuals that had been treated with thymidine-analog NRTIs and were later switched to newer treatments have not always achieved the complete reversal of this phenomenon (see more below). Apart from the key role of thymidine-analog NRTIs, various factors influence the propensity to develop lipoatrophy; it occurs more frequently in men than in women, and the infection-related status (low CD4 levels and/or high HIV-1 RNA levels) is also associated with a higher likelihood of developing lipoatrophy [6].

### 2.2. Lipohypertrophy

Lipohypertrophy in the abdominal area is another common manifestation in PLWH, and it concerns intra-abdominal visceral adipose tissue accumulation. In principle, it is unclear to what extent such alterations have any differential features distinct from abdominal obesity occurring in the general population. Trunk lipohypertrophy was primarily attributed to the presence of PIs as part of the combinatorial antiretroviral treatment patterns. Thus, in the absence of thymidine-based NRTI drugs, the presence of PIs in antiretroviral treatment patterns was shown to increase trunk adiposity and associated metabolic alterations in PLWH, similar to what happens in common visceral obesity [13,14].

### 2.3. Lipomatosis

A third phenomenon characteristic of HIV-associated lipodystrophy is lipomatosis in anatomical areas such as the dorso-cervical region, the pubic area, or other locations [15,16]. The fat accumulation in these regions is entirely different from visceral lipohypertrophy, as it occurs in subcutaneous tissue rather than in visceral areas. Additionally, this accumulation results from a lipomatous process caused by cellular alterations in adipocytes that acquire proliferative properties, shown by the enhanced levels of the “proliferating cell nuclear antigen” (PCNA) and decreased telomere length [17]. In addition to cellular and molecular studies demonstrating these proliferative characteristics, the lipomatous features of these fat depots became particularly evident when the use of enlarged dorso-cervical fat to refill facial lipoatrophy had to be discontinued due to persistent adipose expansion in the cheeks [18]. Moreover, dorso-cervical lipomatosis is associated with a distortion of the white/brown phenotype of adipocytes characterized by the expression of typical brown adipose tissue marker genes such as uncoupling protein-1 (UCP1); however, adipose tissue in lipomatous areas does not acquire functional thermogenic properties [19,20].

### 2.4. Systemic Alterations in HIV-Associated Lipodystrophy

Lipodystrophy in PLWH is associated with important systemic alterations, often including insulin resistance, type 2 diabetes, dyslipidemia, hepatic steatosis, and elevated cardiovascular risk. Various processes associated with HIV-related lipodystrophy contribute to this scenario. Both subcutaneous adipose tissue atrophy and visceral hypertrophy generate local and systemic pro-inflammatory responses [21]. Studies on common obesity have widely indicated that a sustained local pro-inflammatory state in adipose tissue leads to systemic metabolic alterations, such as insulin resistance [22], which is also observed in PLWH affected by lipodystrophy. Alterations in adipose tissue also lead to the disrupted secretion of regulatory adipokines, and abnormally low levels of circulating adiponectin, an anti-inflammatory and insulin-sensitizing factor [23], have long been observed in PLWH [24]. Several in vitro studies have confirmed that first-generation transcriptase inhibitors and PI drugs induce secretion of inflammatory cytokines and suppress the secretion of adiponectin by adipocytes [25,26,27,28]. Various alterations in the leptin levels have been reported in PLWH with lipodystrophy, ranging from abnormally low levels to significantly high levels [29]. This is most likely due to the variable impact on the overall adipose tissue level resulting from the combination of lipoatrophy in subcutaneous areas and lipohypertrophy at the visceral level in each individual.

On the other hand, PLWH with lipodystrophy often exhibit, within the same individual, the harmful consequences of lipotoxicity arising from both the hypotrophy of subcutaneous adipose tissue and the hypertrophy of visceral adipose tissue [30,31]. As is known from the genetic forms of lipoatrophy, an inability to adequately store lipids in adipose tissue promotes ectopic lipid deposition in multiple tissues, impairing their function. A parallel phenomenon occurs as a result of visceral fat hypertrophy; once its expandability limits are reached, lipid spillover into other tissues is also promoted. Thus, an increase in the lipid content in the skeletal muscle of PLWH has been reported, a phenomenon known to contribute to insulin resistance [32]. Hepatic steatosis also frequently appears in PLWH affected by lipodystrophy [33] and may evolve to metabolic dysfunction-associated steatotic liver disease (MASLD). Myocardial lipid accumulation has also been reported in PLWH, even in the absence of abdominal lipohypertrophy [34].

Microbiota dysbiosis, defined as an imbalance in the composition of the gut microbiota, is a well-established contributor to systemic chronic inflammation in obesity, and emerging evidence suggests that similar mechanisms may be present in PLWH. Several studies have reported intestinal dysbiosis and reductions in bacterial alpha diversity in PLWH [35,36]. Notably, bacterial alpha diversity has been shown to correlate with the visceral adipose tissue volume in this population [37]. Furthermore, PLWH exhibit a lower abundance of butyrate-producing bacteria [38], which may impact adipose tissue function, as butyrate is a key short-chain fatty acid known to influence this tissue. Although existing studies are still limited, the specific role of microbiota alterations in driving adipose tissue dysfunction in PLWH and to what extent they affect lipoatrophic and lipohypertrophic alterations in distinct adipose depots remain active areas of investigation.

## 3. Mechanisms Underlying HIV-Associated Lipodystrophy

### 3.1. Antiretroviral Treatment-Driven Effects

The cellular and molecular mechanisms underlying lipoatrophy in PLWH have been extensively studied. In affected patients, reductions in the size and accumulation of fat in subcutaneous adipocytes in lipodystrophic areas are observed, along with apoptosis and cell death [39]. This is accompanied by the infiltration of macrophages and other inflammatory cells [40,41].

First-generation NRTI drugs, especially thymidine analogs (e.g., zidovudine and stavudine), play a key role in lipoatrophy in PLWH. This is not the first case in medicine where a lipoatrophic reaction to a pharmacological treatment has been observed. However, the reported cases of subcutaneous lipoatrophy associated with treatments using insulin, growth hormone analogs, or corticosteroids are typically localized at the injection sites and tend to resolve spontaneously [42]. In contrast, the lipoatrophic effect of thymidine-analog NRTIs is systemic and, as previously mentioned, affects multiple subcutaneous adipose tissue depots across different anatomical sites.

The primary action of zidovudine and stavudine as lipoatrophic agents is attributed to the so-called mitochondrial toxicity. These drugs, along with their ability to inhibit HIV reverse transcriptase, have the collateral effect of inhibiting DNA polymerase-gamma, the enzyme responsible for mitochondrial DNA (mtDNA) replication. Treatment with these NRTIs results in the depletion of mtDNA levels in multiple tissues and organs at a range that is lower than that seen in genetic mtDNA depletion diseases but still biologically relevant [43]. The basis for the tissue-specific susceptibility of adipose tissue to this depletion is not well understood. Mitochondrial dysfunction caused by mtDNA depletion leads to energy deficits, the increased production of reactive oxygen species, and the abnormal proliferation of nonfunctional mitochondria, potentially causing adipocyte death. In vitro studies also demonstrate that these NRTIs inhibit the differentiation of preadipocytes into mature adipocytes by blocking the expression of key adipogenesis genes such as peroxisome proliferator-activated-γ (PPARγ) and others [26,44,45]. As a result, subcutaneous adipose tissue experiences dysfunction in replacing dead adipocytes with new ones, creating the context for lipoatrophy. The importance of mtDNA alterations in lipodystrophy has also been evidenced by the observation of different susceptibilities to lipodystrophy among individuals with different mitochondrial DNA haplogroups [46]. The basis for the particular susceptibility of subcutaneous adipose tissue to develop lipodystrophy remains poorly understood. Although studies on this matter are scarce, it has been reported that mtDNA depletion is similar in subcutaneous and visceral adipose tissues from PLWH, despite the latter not showing atrophy [47].

The precise mechanisms of the other main feature of HIV lipodystrophy, abdominal lipohypertrophy, also have not been fully delineated. The well-known distinct properties of visceral and subcutaneous adipose tissue may involve differential responsiveness to HIV infection and antiretroviral treatment insults. It has been reported that visceral and subcutaneous adipocytes from humans show differential sensitivity to the metabolic alterations elicited by PIs [48]. On the other hand, it cannot be ruled out that a relevant component of fat accumulation in visceral adipose tissue may be secondary to lipoatrophy in peripheral subcutaneous adipose tissue [49]. The impairment in the fat storage capacity in subcutaneous adipose tissue could lead to a redistribution of fat to the visceral compartment, which, for unknown reasons, does not exhibit atrophy in response to mitochondrial toxicity caused by drugs.

As mentioned above, mtDNA depletion cannot explain the differential behavior of visceral and subcutaneous fat in HIV-associated lipodystrophy, but unaffected adipogenesis and a milder induction of pro-inflammatory signaling in visceral relative to subcutaneous fat have been reported [48], which may explain the prevention of fat wasting in visceral adipose tissue.

The exact causes of lipomatosis are also not fully understood. However, the reduction in lipoatrophy associated with avoiding the use of thymidine-analog NRTIs has also led to a decreased occurrence of this phenomenon in PLWH [50]. Importantly, the lipomatous accumulations in the dorso-cervical area and other sites show similar signs of mtDNA depletion as lipoatrophic subcutaneous adipose tissue sites [17,19]. However, other alterations occurring in adipose depots affected by HIV-associated lipodystrophy, such as impaired adipogenesis and enhanced inflammation, do not occur in the enlarged adipose tissues in the dorso-cervical region and other areas [20]. In contrast, lipomatous adipose tissue in PLWH shows the accumulation of the toxic, non-processed form of lamin A, a phenomenon previously reported to take place in genetically determined lipomatosis [51] and in lipoatrophic areas in PLWH [20], so it does not appear to account for the differential behavior of HIV-associated lipodystrophy and lipoatrophic and lipomatous areas.

Subcutaneous adipose tissue in PLWH with lipodystrophy also shows the reduced expression of the micro-RNA-processing enzyme DICER, particularly in the lipomatous dorso-cervical area. This phenomenon is associated with the decreased expression of brown/beige adipose marker genes [52]. DICER dysregulation is associated with the duration of antiretroviral use and, interestingly, correlates with alterations in circulating miRNAs from small extracellular vesicles (enhanced miR-20a-3p and depleted miR-186 and miR-324-5p), which could also contribute to acquired lipodystrophy and associated metabolic and inflammatory perturbations in PLWH [53].

### 3.2. HIV Infection-Related Effects

In addition to antiretroviral treatments, various molecular and cellular events associated with HIV infection also contribute to the development of HIV-associated lipodystrophy. Although HIV-1 does not significantly infect adipocytes [54], it does target immune cells infiltrating adipose tissue, primarily macrophages and lymphocytes [55,56]. Adipose tissue is, in fact, considered a reservoir for HIV-1 in the body, largely due to the infection of these immune cells. One consequence of HIV-1 infection in adipose tissue is the release of inflammatory cytokines. This contributes to local alterations in adipose tissue and can lead to systemic metabolic abnormalities, such as insulin resistance and dyslipidemia [57], features similar to those observed in obesity, a condition characterized by increased local inflammation in adipose tissue. Furthermore, HIV-infected cells secrete extracellular viral-encoded proteins such as Vpr, Tat, and Nef, which can interfere with adipocyte differentiation; promote the expression of pro-inflammatory cytokines; and induce cellular senescence by inhibiting the expression of target genes of PPAR γ, the master regulator of adipogenesis, among other actions [58,59,60,61]. In this way, several alterations in adipogenic and mitochondrial gene expression are already present in the adipose tissue of untreated (naïve) HIV patients [62]. Thus, HIV infection of adipose tissue appears to prime the tissue for increased susceptibility to the structural and functional alterations characteristic of HIV-associated lipodystrophy that occur in response to antiretroviral therapy.

**Figure 2:**
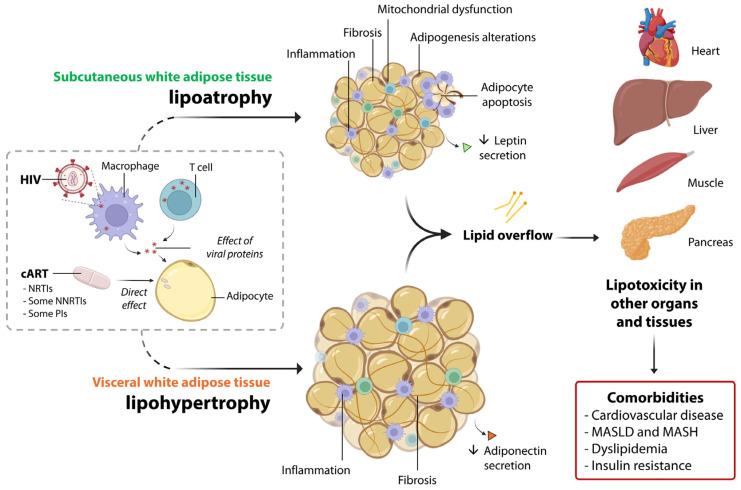
A summary of the main pathophysiological alterations in subcutaneous and visceral white adipose tissue associated with lipodystrophy in PLWH. HIV is present within adipose-resident T cells and macrophages, and interactions between HIV proteins and adipocytes also occur. Additionally, certain drugs used in cART, such as NRTIs, NNRTIs, and PIs, directly affect the molecular functions of adipocytes. Together, these factors contribute to the pathological alterations observed in both the subcutaneous and visceral adipose tissue compartments, which in turn promote lipotoxicity in other organs. This ultimately leads to several systemic comorbidities, such as cardiovascular disease, MASLD, metabolic dysfunction-associated steatohepatitis (MASH), dyslipidemia, and insulin resistance.

Figure 2 summarizes the main molecular actors eliciting lipoatrophy and lipohypertrophy in PLWH, as well as systemic alterations.

## 4. Adipose Tissue Changes Under Modern Antiretroviral Regimens in PLWH

The implementation of last-generation antiretroviral treatment patterns incorporating fewer toxic inhibitors of reverse transcriptase and drugs acting on new targets of HIV biology, e.g., integrase strand transfer inhibitors (INSTIs), has minimized HIV-associated lipoatrophy and lipomatosis. However, soon after the implementation of these treatments in PLWH, increased body weight was observed. Although part of this effect was attributed to the so-called “return to health” state elicited by the initiation of these treatments in PLWH, growing evidence shows specific treatment effects. Increased weight gain has been observed more frequently in PLWH treated with INSTIs and TAF [63,64]. Increased adiposity in response to INSTIs appears to involve the two commonly used INSTI drugs dolutegravir and bictegravir, and it is additive to the weight gain effects elicited by TAF. Increased adiposity occurs when INSTI-based treatments replace older patters of treatment, especially when INSTIs are present in initial treatment regimens after infection [65]. Moreover, some risk factors associated with increased weight gain in PLWH in response to INSTI-containing drug patterns have been identified: sex (women are more prone to weight gain than men), black ethnicity, and aging. Weight gain in PLWH under INSTI/TAF-based treatments results from generalized increases in adipose tissue mass in both the subcutaneous and trunk areas [66]. Therefore, the alterations elicited by last-generation drug treatments in PLWH are more reminiscent of obesity than lipodystrophy [67], at least based on what is known so far.

Some initial hypotheses suggested that INSTIs could affect the hypothalamic melanocortin system and thereby interfere with satiety mechanisms, leading to increased caloric intake and subsequent weight gain. This was based on some data indicating that INSTIs may interfere with the melanocortin-4 receptor (MC4R) [68]. However, other reports do not support this proposition, as the doses of INSTIs that interfere with the MC4R are higher than those found in treated patients [69]. In fact, there are still no objective studies that evaluate the effects of INSTIs on food intake [69].

Several experimental studies on human adipocytes [70,71], though not all [72], suggest that the integrase inhibitor dolutegravir has direct pro-adipogenic effects. A possible explanation for this discrepancy lies in the different sources of adipocytes used in vitro: some studies [70,71] utilized adipocytes differentiated from primary cultures of adipose tissue stem cells obtained from healthy women, while another study [72] used cells from an infant with Simpson–Golabi–Behmel syndrome. Despite these differences, multiple studies consistently report that dolutegravir suppresses the expression of brown/beige adipose tissue markers and reduces adiponectin levels in adipocytes [70,71,72,73]. Furthermore, dolutegravir has been associated with increased fibrosis, mitochondrial dysfunction, and insulin resistance in adipocytes [70,71]. Comparable alterations—including increased fibrosis, enlarged adipocytes, and the upregulated expression of adipogenic genes—have also been observed in subcutaneous and visceral adipose tissues from macaques treated with INSTIs, both in the absence [71] and presence [70] of concomitant simian immunodeficiency virus infection, with the latter condition showing a particularly marked impairment in beige adipose tissue. Fibrosis induction has also been observed in both adipose depots in PLWH treated with INSTIs [71]. Taken together, these findings consistently point to adverse structural and functional changes in adipose tissue linked to INSTI treatments, despite the inherent limitations of studies using in vitro and animal models.

The in vitro and in vivo evidence also reveals complex and sometimes contrasting effects of TDF and TAF on adipose tissue. In vitro studies using mouse 3T3-L1 adipocytes showed that TAF and TDF inhibited adipogenesis alone and in combination with INSTIs [74], whereas other studies using human adipocytes showed that both drugs moderate the anti-adipogenic effect of the INSTI dolutegravir [73]. However, the in vivo effects appear more divergent, with TAF being associated with greater weight gain and fat accumulation than TDF in both clinical and animal models [75,76]. Several factors may explain these differences. First, the direct effects of these drugs on adipocytes may differ from their systemic effects when administered in vivo. Second, their interactions with other antiretroviral drugs in combination regimens likely play an essential role. Third, the metabolic context, including the HIV infection status, immune activation, and baseline metabolic health, may significantly modify the effects of these drugs on adipose tissue.

Another aspect is how the last-generation antiretroviral treatments for PLWH affect systemic metabolism. There have been some indications that INSTIs may be associated with positive effects on metabolism, such as improved dyslipidemia and insulin sensitivity [77], at least compared to non-INSTI-based treatment regimens. However, other studies have shown deleterious effects on metabolism, which may be due to the actions of the drugs or may be indirectly caused by visceral obesity that appears in patients. This is the case for hypoadiponectinemia in PLWH treated with antiretroviral patterns that include dolutegravir [78]. Studies examining the impacts of antiretroviral treatments, including INSTIs such as dolutegravir, on cardiometabolic parameters have found unfavorable changes, such as increased HbA1c and blood pressure; a higher incidence of cardiovascular disease; and elevated risks for several cardiometabolic outcomes, including congestive heart failure, myocardial infarction, and lipid disorders. These effects are especially evident in cases of long-term treatment with INSTIs combined with TAF [79,80,81]. However, it remains unclear whether specific alterations in adipose tissue caused by antiretroviral drugs, beyond the unhealthy consequences of increased fat mass, contribute to cardiovascular risk.

## 5. Conclusions: Current Challenges for Management of Healthy Adipose Tissue in PLWH

The current clinical concerns related to HIV-associated lipodystrophy manifest in several ways. The avoidance of thymidine-based NRTIs worldwide has decreased the prevalence of lipoatrophy in PLWH. However, a significant number of PLWH who were treated with first-generation NRTIs and developed lipoatrophy did not recover after switching to a treatment based on last-generation antiretroviral drugs. Among the patients in the APROCO cohort previously treated with thymidine-analog NRTIs, 22% continue to experience mixed lipoatrophy/lipohypertrophy and elevated insulin resistance rates, even after transitioning to modern antiretroviral regimens [82]. Visceral lipohypertrophy often persists in PLWH who have had long-term exposure to thymidine-analog NRTIs. Each year of cumulative exposure to first-generation NRTIs is associated with an increase in visceral fat of 3.7 cm^2^ after the discontinuation of these drugs [83]. The persistent signs of lipodystrophy and associated metabolic alterations that occur even after the discontinuation of more lipodystrophy-prone drugs are especially common in PLWH who started treatment during childhood. Increased waist circumference is nearly twice as prevalent in adults with HIV who were exposed to thymidine NRTIs during childhood [84].

Second, although visceral obesity remains the primary adipose alteration in PLWH undergoing contemporary antiretroviral therapy, a recent report revealed that the subcutaneous fat transcriptome in patients with more visceral adipose tissue shows the increased expression of genes associated with the extracellular matrix and inflammation, along with the reduced expression of genes related to lipolysis and fatty acid metabolism [85]. This highlights the current persistence of complex alterations in different adipose depots in PLWH.

Recently, novel aspects relating adipose tissue dysfunction and conditions compromising health in PLWH are emerging. It has long been known that PLWH show an increased risk of diastolic dysfunction. Recent data indicate that epicardial adipose tissue, a fat depot closely associated with the myocardium and known to be involved in cardiac function [86], is enlarged in PLWH. This is associated with diastolic dysfunction, regardless of the HIV serostatus and viral suppression [87,88]. Increased pericoronary adipose density has been found to be independently associated with the prevalence and severity of coronary plaque in PLWH [89]. There are also data indicating that PLWH show signs of inflammation in perivascular fat, the type of adipose tissue coating vessels. This inflammation could be involved in the microvascular disease that often occurs in PLWH [90]. As such, the potential roles of altered epicardiac, pericoronary, and perivascular adipose tissue in cardiovascular function among PLWH remain speculative and are based on observed correlations and the more established impacts of these fat depots on cardiac and vascular pathophysiology in the general population. In any case, such findings provide further evidence of the influence of distorted adipose tissue on health conditions in PLWH.

Treatment strategies based on lifestyle changes are increasingly considered to be relevant for mitigating adipose tissue alterations and systemic cardiometabolic disturbances in PLWH. When overt lipodystrophy was highly prevalent, some data indicated that adhesion to a Mediterranean diet and supplementation with omega-3 polyunsaturated fatty acids ameliorated systemic parameters, including inflammation [91,92]. Notably, an intervention based on the Mediterranean diet led to a marked increase in the relative abundance of Bifidobacterium spp., which is linked to a microbiota profile indicative of beneficial immunometabolic outcomes [93]. In the current context of increased obesity in PLWH under treatments including INSTIs, even in the absence of accurate specific studies, clinical practices recommend lifestyle modifications (a decrease in caloric intake and an increase in exercise habits) as in the first-line treatment approach for common obesity.

On the other hand, several recent studies have shown that, in PLWH, GLP-1 receptor agonists commonly used to treat obesity and type 2 diabetes (e.g., semaglutide) produce a degree of weight loss similar to that observed in the general population [94], along with reductions in both visceral and subcutaneous adipose tissue [95]. A clinical trial investigating the effects of semaglutide in overweight PLWH found a significant reduction in abdominal visceral adipose tissue, which was the most pronounced effect [96]. These findings suggest that incretin analogs, currently used to treat common obesity and type 2 diabetes, may also hold promise as therapeutic agents for managing visceral lipohypertrophy and the associated metabolic dysregulation in PLWH [97].

In summary, recent data underscore the ongoing vulnerability of PLWH to changes in adipose tissue. Although the current alterations in adipose tissue among PLWH may not be as severe as traditional HIV-related lipodystrophy, they can still have significant health implications. To improve the well-being of PLWH, it is essential to continue refining antiretroviral treatment strategies and to monitor potential adipose tissue changes as part of comprehensive metabolic health management.

## Abbreviations

The following abbreviations are used in this manuscript:

AIDS	acquired immunodeficiency syndrome
cART	combined antiretroviral therapy
GLP-1	glucagon-like peptide-1
HIV	human immunodeficiency virus
INSTI	integrase strand transfer inhibitor
MASLD	metabolic dysfunction-associated steatotic liver disease
MASH	metabolic dysfunction-associated steatohepatitis
MC4R	melanocortin-4 receptor
mtDNA	mitochondrial DNA
NRTI	nucleotide analog reverse transcriptase inhibitor
NNRTI	non-nucleoside analog reverse transcriptase inhibitor
PCNA	proliferating cell nuclear antigen
PI	protease inhibitor
PLWH	people living with HIV
PPARγ	peroxisome proliferator-activated-γ
TAF	tenofovir alafenamide
TDF	tenofovir difumarate
UCP1	uncoupling protein-1

## References

[1] Giralt M., Domingo P., Villarroya F. (2011). Adipose tissue biology and HIV-infection. Best Pract. Res. Clin. Endocrinol. Metab..

[2] Koethe J.R., Lagathu C., Lake J.E., Domingo P., Calmy A., Falutz J., Brown T.T., Capeau J. (2020). HIV and antiretroviral therapy-related fat alterations. Nat. Rev. Dis. Primers.

[3] Ramirez Bustamante C.E., Agarwal N., Cox A.R., Hartig S.M., Lake J.E., Balasubramanyam A. (2024). Adipose Tissue Dysfunction and Energy Balance Paradigms in People Living with HIV. Endocr. Rev..

[4] Capeau J., Lagathu C., Béréziat V., Fève B. (2021). Recent data on adipose tissue, insulin resistance, diabetes and dyslipidaemia in antiretroviral therapy controlled HIV-infected persons. Curr. Opin. HIV AIDS.

[5] Tilhet L., Rouanet M., Henry A.S., Pop A., Claudic Y., Saraoui W., Nachaoui H., Hu W. (2025). Current status and perspectives in the treatment of facial lipoatrophy in HIV-positive patients in 2024. Ann. Chir. Plast. Esthet..

[6] Domingo P., Estrada V., López-Aldeguer J., Villaroya F., Martínez E. (2012). Fat redistribution syndromes associated with HIV-1 infection and combination antiretroviral therapy. AIDS Rev..

[7] Carr A., Law M. (2003). HIV Lipodystrophy Case Definition Study Group. An objective lipodystrophy severity grading scale derived from the lipodystrophy case definition score. J. Acquir. Immune Defic. Syndr..

[8] Mallon P.W., Miller J., Cooper D.A., Carr A. (2003). Prospective evaluation of the effects of antiretroviral therapy on body composition in HIV-1-infected men starting therapy. AIDS.

[9] Martin A., Mallon P.W. (2005). Therapeutic approaches to combating lipoatrophy: Do they work?. J. Antimicrob. Chemother..

[10] Glidden D.V., Mulligan K., McMahan V., Anderson P.L., Guanira J., Chariyalertsak S., Buchbinder S.P., Bekker L.G., Schechter M., Grinsztejn B. (2018). Metabolic Effects of Preexposure Prophylaxis with Coformulated Tenofovir Disoproxil Fumarate and Emtricitabine. Clin. Infect. Dis..

[11] Martínez E., Ribera E., Clotet B., Estrada V., Sanz J., Berenguer J., Rubio R., Pulido F., Larrousse M., Curran A. (2015). Switching from zidovudine/lamivudine to tenofovir/emtricitabine improves fat distribution as measured by fat mass ratio. HIV Med..

[12] Capeau J., Lagathu C., Béréziat V. (2024). Recent data on the role of antiretroviral therapy in weight gain and obesity in persons living with HIV. Curr. Opin. HIV AIDS.

[13] Ferrer E., Navarro A., Curto J., Medina P., Rozas N., Barrera G., Saumoy M., Tiraboschi J.M., Gomez C., Podzamczer D. (2014). Long-term fat redistribution in ARV-naïve HIV+ patients initiating a non-thymidine containing regimen in clinical practice. J. Int. AIDS Soc..

[14] Grant P.M., Kitch D., McComsey G.A., Collier A.C., Bartali B., Koletar S.L., Erlandson K.M., Lake J.E., Yin M.T., Melbourne K. (2016). Long-term body composition changes in antiretroviral-treated HIV-infected individuals. AIDS.

[15] Lo J.C., Mulligan K., Tai V.W., Algren H., Schambelan M. (1998). “Buffalo hump” in men with HIV-1 infection. Lancet.

[16] Guaraldi G., Orlando G., Squillace N., Roverato A., De Fazio D., Vandelli M., Nardini G., Beghetto B., De Paola M., Esposito R. (2007). Prevalence of and risk factors for pubic lipoma development in HIV-infected persons. J. Acquir. Immune Defic. Syndr..

[17] Cereijo R., Gallego-Escuredo J.M., Moure R., Villarroya J., Domingo J.C., Fontdevila J., Martínez E., Gutiérrez M.M., Mateo M.G., Giralt M. (2015). The Molecular Signature of HIV-1-Associated Lipomatosis Reveals Differential Involvement of Brown and Beige/Brite Adipocyte Cell Lineages. PLoS ONE.

[18] Guaraldi G., De Fazio D., Orlando G., Murri R., Wu A., Guaraldi P., Esposito R. (2005). Facial lipohypertrophy in HIV-infected subjects who underwent autologous fat tissue transplantation. Clin. Infect. Dis..

[19] Guallar J.P., Gallego-Escuredo J.M., Domingo J.C., Alegre M., Fontdevila J., Martínez E., Hammond E.L., Domingo P., Giralt M., Villarroya F. (2008). Differential gene expression indicates that ‘buffalo hump’ is a distinct adipose tissue disturbance in HIV-1-associated lipodystrophy. AIDS.

[20] Béréziat V., Cervera P., Le Dour C., Verpont M.C., Dumont S., Vantyghem M.C., Capeau J., Vigouroux C., Lipodystrophy Study Group (2011). LMNA mutations induce a non-inflammatory fibrosis and a brown fat-like dystrophy of enlarged cervical adipose tissue. Am. J. Pathol..

[21] Bourgeois C., Gorwood J., Olivo A., Le Pelletier L., Capeau J., Lambotte O., Béréziat V., Lagathu C. (2021). Contribution of Adipose Tissue to the Chronic Immune Activation and Inflammation Associated with HIV Infection and Its Treatment. Front. Immunol..

[22] Ghaben A.L., Scherer P.E. (2019). Adipogenesis and metabolic health. Nat. Rev. Mol. Cell. Biol..

[23] Kadowaki T., Yamauchi T., Kubota N., Hara K., Ueki K., Tobe K. (2006). Adiponectin and adiponectin receptors in insulin resistance, diabetes, and the metabolic syndrome. J. Clin. Investig..

[24] Tong Q., Sankal J.L., Hadigan C.M., Tan G., Rosenberg E.S., Kanki P.J., Grinspoon S.K., Hotamisligil G.S. (2003). Regulation of adiponectin in human immunodeficiency virus-infected patients: Relationship to body composition and metabolic in-dices. J. Clin. Endocrinol. Metab..

[25] Lagathu C., Bastard J.P., Auclair M., Maachi M., Kornprobst M., Capeau J., Caron M. (2004). Antiretroviral drugs with adverse effects on adipocyte lipid metabolism and survival alter the expression and secretion of proinflammatory cytokines and adiponectin in vitro. Antivir. Ther..

[26] Lagathu C., Eustace B., Prot M., Frantz D., Gu Y., Bastard J.P., Maachi M., Azoulay S., Briggs M., Caron M. (2007). Some HIV antiretrovirals increase oxidative stress and alter chemokine, cytokine or adiponectin production in human adipocytes and macrophages. Antivir. Ther..

[27] Jones S.P., Janneh O., Back D.J., Pirmohamed M. (2005). Altered adipokine response in murine 3T3-F442A adipocytes treated with protease inhibitors and nucleoside reverse transcriptase inhibitors. Antivir. Ther..

[28] Gallego-Escuredo J.M., Del Mar Gutierrez M., Diaz-Delfin J., Domingo J.C., Mateo M.G., Domingo P., Giralt M., Villarroya F. (2010). Differential effects of efavirenz and lopinavir/ritonavir on human adipocyte differentiation, gene expression and release of adipokines and pro-inflammatory cytokines. Curr. HIV Res..

[29] Tiliscan C., Aramă V., Mihăilescu R., Munteanu D.I., Streinu-Cercel A., Ion D.A., Rădulescu M.A., Popescu C., Lobodan A.E., Negru A.R. (2015). Leptin expression in HIV-infected patients during antiretroviral therapy. Germs.

[30] Giralt M., Díaz-Delfín J., Gallego-Escuredo J.M., Villarroya J., Domingo P., Villarroya F. (2010). Lipotoxicity on the basis of metabolic syndrome and lipodystrophy in HIV-1-infected patients under antiretroviral treatment. Curr. Pharm. Des..

[31] Villarroya F., Domingo P., Giralt M. (2010). Drug-induced lipotoxicity: Lipodystrophy associated with HIV-1 infection and antiretroviral treatment. Biochim. Biophys. Acta.

[32] Torriani M., Thomas B.J., Barlow R.B., Librizzi J., Dolan S., Grinspoon S. (2006). Increased intramyocellular lipid accumulation in HIV-infected women with fat redistribution. J. Appl. Physiol..

[33] Sutinen J., Häkkinen A.M., Westerbacka J., Seppälä-Lindroos A., Vehkavaara S., Halavaara J., Järvinen A., Ristola M., Yki-Järvinen H. (2002). Increased fat accumulation in the liver in HIV-infected patients with antiretroviral therapy-associated lipodystrophy. AIDS.

[34] Neilan T.G., Nguyen K.L., Zaha V.G., Chew K.W., Morrison L., Ntusi N.A.B., Toribio M., Awadalla M., Drobni Z.D., Nelson M.D. (2020). Myocardial Steatosis Among Antiretroviral Therapy-Treated People with Human Immunodeficiency Virus Participating in the REPRIEVE Trial. J. Infect. Dis..

[35] Tuddenham S.A., Koay W.L.A., Zhao N., White J.R., Ghanem K.G., Sears C.L., HIV Microbiome Re-analysis Consortium (2020). The Impact of Human Immunodeficiency Virus Infection on Gut Microbiota α-Diversity: An Individual-level Meta-analysis. Clin. Infect. Dis..

[36] Brenchley J.M., Price D.A., Schacker T.W., Asher T.E., Silvestri G., Rao S., Kazzaz Z., Bornstein E., Lambotte O., Altmann D. (2006). Microbial translocation is a cause of systemic immune activation in chronic HIV infection. Nat. Med..

[37] Gogokhia L., Taur Y., Juluru K., Yagan N., Zhu Y.S., Pamer E., Glesby M.J. (2020). Intestinal Dysbiosis and Markers of Systemic Inflammation in Viscerally and Generally Obese Persons Living with HIV. J. Acquir. Immune Defic. Syndr..

[38] Dillon S.M., Kibbie J., Lee E.J., Guo K., Santiago M.L., Austin G.L., Gianella S., Landay A.L., Donovan A.M., Frank D.N. (2017). Low abundance of colonic butyrate-producing bacteria in HIV infection is associated with microbial translocation and immune activation. AIDS.

[39] Lloreta J., Domingo P., Pujol R.M., Arroyo J.A., Baixeras N., Matias-Guiu X., Gilaberte M., Sambeat M.A., Serrano S. (2002). Ultra-structural features of highly active antiretroviral therapy-associated partial lipodystrophy. Virchows Arch..

[40] Jan V., Cervera P., Maachi M., Baudrimont M., Kim M., Vidal H., Girard P.M., Levan P., Rozenbaum W., Lombès A. (2004). Altered fat differentiation and adipocytokine expression are interrelated and linked to morphological changes and insulin resistance in HIV-1-infected lipodystrophic patients. Antivir. Ther..

[41] Guaraldi G., Luzi K., Bellistrì G.M., Zona S., Domingues da Silva A.R., Bai F., Garlassi E., Marchetti G., Capeau J., Monforte A.D. (2013). CD8 T-cell activation is associated with lipodystrophy and visceral fat accumulation in antiretroviral therapy-treated virologically suppressed HIV-infected patients. J. Acquir. Immune Defic. Syndr..

[42] Giralt M., Villarroya F., Araujo-Vilar D., Huhyaniemi I., Martini L. (2019). Lipodystrophies. Encyclopedia of Endocrine Diseases.

[43] Villarroya F., Domingo P., Giralt M. (2005). Lipodystrophy associated with highly active anti-retroviral therapy for HIV infection: The adipocyte as a target of anti-retroviral-induced mitochondrial toxicity. Trends Pharmacol. Sci..

[44] Caron M., Auclair M., Lagathu C., Lombès A., Walker U.A., Kornprobst M., Capeau J. (2004). The HIV-1 nucleoside reverse transcriptase inhibitors stavudine and zidovudine alter adipocyte functions in vitro. AIDS.

[45] Stankov M.V., Lücke T., Das A.M., Schmidt R.E., Behrens G.M. (2010). Mitochondrial DNA depletion and respiratory chain activity in primary human subcutaneous adipocytes treated with nucleoside analogue reverse transcriptase inhibitors. Antimicrob. Agents Chemother..

[46] De Luca A., Nasi M., Di Giambenedetto S., Cozzi-Lepri A., Pinti M., Marzocchetti A., Mussini C., Fabbiani M., Bracciale L., Cauda R. (2012). Mitochondrial DNA haplogroups and incidence of lipodystrophy in HIV-infected patients on long-term antiretroviral therapy. J. Acquir. Immune Defic. Syndr..

[47] Gallego-Escuredo J.M., Villarroya J., Domingo P., Targarona E.M., Alegre M., Domingo J.C., Villarroya F., Giralt M. (2013). Differentially altered molecular signature of visceral adipose tissue in HIV-1-associated lipodystrophy. J. Acquir. Immune Defic. Syndr..

[48] Kovsan J., Osnis A., Maissel A., Mazor L., Tarnovscki T., Hollander L., Ovadia S., Meier B., Klein J., Bashan N. (2009). Depot-specific adipocyte cell lines reveal differential drug-induced responses of white adipocytes--relevance for partial lipodystrophy. Am. J. Physiol. Endocrinol. Metab..

[49] Villarroya F., Domingo P., Giralt M. (2007). Lipodystrophy in HIV 1-infected patients: Lessons for obesity research. Int. J. Obes..

[50] Srinivasa S., Grinspoon S.K. (2014). Metabolic and body composition effects of newer antiretrovirals in HIV-infected patients. Eur. J. Endocrinol..

[51] Shackleton S., Lloyd D.J., Jackson S.N., Evans R., Niermeijer M.F., Singh B.M., Schmidt H., Brabant G., Kumar S., Durrington P.N. (2000). LMNA, encoding lamin A/C, is mutated in partial lipodystrophy. Nat. Genet..

[52] Torriani M., Srinivasa S., Fitch K.V., Thomou T., Wong K., Petrow E., Kahn C.R., Cypess A.M., Grinspoon S.K. (2016). Dysfunctional Subcutaneous Fat with Reduced Dicer and Brown Adipose Tissue Gene Expression in HIV-Infected Patients. J. Clin. Endocrinol. Metab..

[53] Srinivasa S., Garcia-Martin R., Torriani M., Fitch K.V., Carlson A.R., Kahn C.R., Grinspoon S.K. (2021). Altered pattern of circulating miRNAs in HIV lipodystrophy perturbs key adipose differentiation and inflammation pathways. JCI Insight.

[54] Munier S., Borjabad A., Lemaire M., Mariot V., Hazan U. (2003). In vitro infection of human primary adipose cells with HIV-1: A reassessment. AIDS.

[55] Couturier J., Suliburk J.W., Brown J.M., Luke D.J., Agarwal N., Yu X., Nguyen C., Iyer D., Kozinetz C.A., Overbeek P.A. (2015). Human adipose tissue as a reservoir for memory CD4+ T cells and HIV. AIDS.

[56] Damouche A., Lazure T., Avettand-Fènoël V., Huot N., Dejucq-Rainsford N., Satie A.P., Mélard A., David L., Gommet C., Ghosn J. (2015). Adipose Tissue Is a Neglected Viral Reservoir and an Inflammatory Site during Chronic HIV and SIV Infection. PLoS Pathog..

[57] Couturier J., Lewis D.E. (2018). HIV Persistence in Adipose Tissue Reservoirs. Curr. HIV/AIDS Rep..

[58] Gorwood J., Bourgeois C., Mantecon M., Atlan M., Pourcher V., Pourcher G., Le Grand R., Desjardins D., Fève B., Lambotte O. (2019). Impact of HIV/simian immunodeficiency virus infection and viral proteins on adipose tissue fibrosis and adipogenesis. AIDS.

[59] Gorwood J., Ejlalmanesh T., Bourgeois C., Mantecon M., Rose C., Atlan M., Desjardins D., Le Grand R., Fève B., Lambotte O. (2020). SIV Infection and the HIV Proteins Tat and Nef Induce Senescence in Adipose Tissue and Human Adipose Stem Cells, Resulting in Adipocyte Dysfunction. Cells.

[60] Agarwal N., Balasubramanyam A. (2014). Viral mechanisms of adipose dysfunction: Lessons from HIV-1 Vpr. Adipocyte.

[61] Díaz-Delfín J., Domingo P., Wabitsch M., Giralt M., Villarroya F. (2012). HIV-1 Tat protein impairs adipogenesis and induces the expression and secretion of proinflammatory cytokines in human SGBS adipocytes. Antivir. Ther..

[62] Giralt M., Domingo P., Guallar J.P., Rodriguez de la Concepción M.L., Alegre M., Domingo J.C., Villarroya F. (2006). HIV-1 infection alters gene expression in adipose tissue, which contributes to HIV- 1/HAART-associated lipodystrophy. Antivir. Ther..

[63] Sax P.E., Erlandson K.M., Lake J.E., Mccomsey G.A., Orkin C., Esser S., Brown T.T., Rockstroh J.K., Wei X., Carter C.C. (2020). Weight Gain Following Initiation of Antiretroviral Therapy: Risk Factors in Randomized Comparative Clinical Trials. Clin. Infect. Dis..

[64] Venter W.D.F., Moorhouse M., Sokhela S., Fairlie L., Mashabane N., Masenya M., Serenata C., Akpomiemie G., Qavi A., Chandiwana N. (2019). Dolutegravir plus Two Different Prodrugs of Tenofovir to Treat HIV. N. Engl. J. Med..

[65] Grabar S., Potard V., Piroth L., Abgrall S., Bernard L., Allavena C., Caby F., de Truchis P., Duvivier C., Enel P. (2023). Striking differences in weight gain after cART initiation depending on early or advanced presentation: Results from the ANRS CO4 FHDH cohort. J. Antimicrob. Chemother..

[66] Lake J.E., Wu K., Bares S.H., Debroy P., Godfrey C., Koethe J.R., McComsey G.A., Palella F.J., Tassiopoulos K., Erlandson K.M. (2020). Risk Factors for Weight Gain Following Switch to Integrase Inhibitor-Based Antiretroviral Therapy. Clin. Infect. Dis..

[67] Bailin S.S., Koethe J.R., Rebeiro P.F. (2024). The pathogenesis of obesity in people living with HIV. Curr. Opin. HIV AIDS.

[68] Domingo P., Villarroya F., Giralt M., Domingo J.C. (2020). Potential role of the melanocortin signaling system interference in the excess weight gain associated to some antiretroviral drugs in people living with HIV. Int. J. Obes..

[69] Chandiwana N.C., Siedner M.J., Marconi V.C., Hill A., Ali M.K., Batterham R.L., Venter W.D.F. (2024). Weight Gain After HIV Therapy Initiation: Pathophysiology and Implications. J. Clin. Endocrinol. Metab..

[70] Ngono Ayissi K., Gorwood J., Le Pelletier L., Bourgeois C., Beaupère C., Auclair M., Foresti R., Motterlini R., Atlan M., Bar-rail-Tran A. (2022). Inhibition of Adipose Tissue Beiging by HIV Integrase Inhibitors, Dolutegravir and Bictegravir, Is Associated with Adipocyte Hypertrophy, Hypoxia, Elevated Fibrosis, and Insulin Resistance in Simian Adipose Tissue and Human Adipocytes. Cells.

[71] Gorwood J., Bourgeois C., Pourcher V., Pourcher G., Charlotte F., Mantecon M., Rose C., Morichon R., Atlan M., Le Grand R. (2020). The Integrase Inhibitors Dolutegravir and Raltegravir Exert Proadipogenic and Profibrotic Effects and Induce Insulin Resistance in Human/Simian Adipose Tissue and Human Adipocytes. Clin. Infect. Dis..

[72] Domingo P., Quesada-López T., Villarroya J., Cairó M., Gutierrez M.D.M., Mateo M.G., Mur I., Corbacho N., Domingo J.C., Villarroya F. (2022). Differential effects of dolutegravir, bictegravir and raltegravir in adipokines and inflammation markers on human adipocytes. Life Sci..

[73] Quesada-López T., Cereijo R., Blasco-Roset A., Mestres-Arenas A., Prieto P., Domingo J.C., Villarroya F., Domingo P., Giralt M. (2024). Divergent effects of the antiretroviral drugs, dolutegravir, tenofovir alafenamide, and tenofovir disoproxil fumarate, on human adipocyte function. Biochem. Pharmacol..

[74] Perna A., Carleo M.A., Mascolo S., Guida A., Contieri M., Sellitto C., Hay E., De Blasiis P., Lucariello A., Guerra G. (2023). Adipocyte differentiation of 3T3-L1 cells under tenofovir alafenamide, tenofovir disoproxil fumarate, and integrase strand transfer inhibitors selective challenge: An in-vitro model. AIDS.

[75] Hocqueloux L., Menard A., Arvieux C., Joly V., Becker A., Chéret A., Duvivier C., Cabié A., Delpierre C., Allavena C. (2023). Weight gain following the single substitution of tenofovir disoproxil fumarate by tenofovir alafenamide in HIV-infected people from the French Dat’AIDS cohort: A propensity score-matched analysis. HIV Med..

[76] Dulion B., Olali A.Z., Patel N., Virdi A.K., Naqib A., Wallace J., Ross R.D. (2025). Tenofovir alafenamide promotes weight gain and impairs fatty acid metabolism-related signaling pathways in visceral fat tissue compared to tenofovir disoproxil fumarate. Antivir. Res..

[77] Milic J., Renzetti S., Ferrari D., Barbieri S., Menozzi M., Carli F., Dolci G., Ciusa G., Mussini C., Calza S. (2022). Relationship between weight gain and insulin resistance in people living with HIV switching to integrase strand transfer inhibitors-based regimens. AIDS.

[78] González-Cordón A., Assoumou L., Moyle G., Waters L., Johnson M., Domingo P., Fox J., Stellbrink H.J., Guaraldi G., Masiá M. (2021). Switching from boosted PIs to dolutegravir decreases soluble CD14 and adiponectin in high cardiovascular risk people living with HIV. J. Antimicrob. Chemother..

[79] Summers N.A., Lahiri C.D., Angert C.D., Aldredge A., Mehta C.C., Ofotokun I., Kerchberger A.M., Gustafson D., Weiser S.D., Kassaye S. (2020). Metabolic Changes Associated with the Use of Integrase Strand Transfer Inhibitors Among Virally Controlled Women. J. Acquir. Immune Defic. Syndr..

[80] Neesgaard B., Greenberg L., Miró J.M., Grabmeier-Pfistershammer K., Wandeler G., Smith C., De Wit S., Wit F., Pelchen-Matthews A., Mussini C. (2022). Associations between integrase strand-transfer inhibitors and cardiovascular disease in people living with HIV: A multicentre prospective study from the RESPOND cohort consortium. Lancet HIV.

[81] Rebeiro P.F., Emond B., Rossi C., Bookhart B.K., Shah A., Caron-Lapointe G., Lafeuille M.H., Donga P. (2023). Incidence of cardiometabolic outcomes among people living with HIV-1 initiated on integrase strand transfer inhibitor versus non-integrase strand transfer inhibitor antiretroviral therapies: A retrospective analysis of insurance claims in the United States. J. Int. AIDS Soc..

[82] Bastard J.P., Couffignal C., Fellahi S., Bard J.M., Mentre F., Salmon D., Katlama C., Raffi F., Leport C., Capeau J. (2019). Diabetes and dyslipidaemia are associated with oxidative stress independently of inflammation in long-term antiretroviral-treated HIV-infected patients. Diabetes Metab..

[83] Gelpi M., Afzal S., Lundgren J., Ronit A., Roen A., Mocroft A., Gerstoft J., Lebech A.M., Lindegaard B., Kofoed K.F. (2018). Higher Risk of Abdominal Obesity, Elevated Low-Density Lipoprotein Cholesterol, and Hypertriglyceridemia, but not of Hypertension, in People Living with Human Immunodeficiency Virus (HIV): Results From the Copenhagen Comorbidity in HIV Infection Study. Clin. Infect. Dis..

[84] Arrive E., Viard J.P., Salanave B., Dollfus C., Matheron S., Reliquet V., Arezes E., Nailler L., Vigouroux C., Warszawski J. (2018). Metabolic risk factors in young adults infected with HIV since childhood compared with the general population. PLoS ONE.

[85] Bailin S.S., Gabriel C.L., Gangula R.D., Hannah L., Nair S., Carr J.J., Terry J.G., Silver H.J., Simmons J.D., Mashayekhi M. (2024). Single-Cell Analysis of Subcutaneous Fat Reveals Profibrotic Cells That Correlate with Visceral Adiposity in HIV. J. Clin. Endocrinol. Metab..

[86] Janssen-Telders C., Eringa E.C., de Groot J.R., de Man F.S., Handoko M.L. (2025). The role of epicardial adipose tissue remodelling in heart failure with preserved ejection fraction. Cardiovasc. Res..

[87] Han W.M., Apornpong T., Tumkosit M., Avihingsanon A., Chattranukulchai P. (2024). Epicardial fat tissue and diastolic dysfunction in both men and women with HIV. AIDS.

[88] Goldberg R.L., Peterson T.E., Haberlen S.A., Witt M.D., Palella F.J., Magnani J.W., Brown T.T., Lake J.E., Lima J.A.C., Budoff M.J. (2024). Response to “Epicardial fat tissue and diastolic dysfunction in both men and women with HIV”. AIDS.

[89] Foldyna B., Mayrhofer T., Zanni M.V., Lyass A., Barve R., Karady J., McCallum S., Burdo T.H., Fitch K.V., Paradis K. (2023). Pericoronary Adipose Tissue Density, Inflammation, and Subclinical Coronary Artery Disease Among People with HIV in the REPRIEVE Cohort. Clin. Infect. Dis..

[90] Wilcox C.S., Herbert C., Wang C., Ma Y., Sun P., Li T., Verbesey J., Kumar P., Kassaye S., Welch W.J. (2024). Signals From Inflamed Perivascular Adipose Tissue Contribute to Small-Vessel Dysfunction in Women with Human Immunodeficiency Virus. J. Infect. Dis..

[91] Basta D., Latinovic O.S., Tagaya Y., Silvestri G. (2024). Potential Advantages of a Well-balanced Nutrition Regimen for People Living with Human Immunodeficiency Virus Type-1. J. AIDS HIV Treat..

[92] Domingo P., Fernández I., Gallego-Escuredo J.M., Torres F., Gutierrez M.D.M., Mateo M.G., Villarroya J., Giralt M., Vidal F., Villarroya F. (2018). Effects of docosahexanoic acid on metabolic and fat parameters in HIV-infected patients on cART: A randomized, double-blind, placebo-controlled study. Clin. Nutr..

[93] Pastor-Ibáñez R., Blanco-Heredia J., Etcheverry F., Sánchez-Palomino S., Díez-Fuertes F., Casas R., Navarrete-Muñoz M.Á., Castro-Barquero S., Lucero C., Fernández I. (2021). Adherence to a Supplemented Mediterranean Diet Drives Changes in the Gut Microbiota of HIV-1-Infected Individuals. Nutrients.

[94] Haidar L., Crane H.M., Nance R.M., Webel A., Ruderman S.A., Whitney B.M., Willig A.L., Napravnik S., Mixson L.S., Leong C. (2024). Weight loss associated with semaglutide treatment among people with HIV. AIDS.

[95] Lee D., Capeau J. (2024). Is the GLP-1 receptor agonist, semaglutide, a good option for weight loss in persons with HIV?. AIDS.

[96] Eckard A.R., Wu Q., Sattar A., Ansari-Gilani K., Labbato D., Foster T., Fletcher A.A., Adekunle R.O., McComsey G.A. (2024). Once-weekly semaglutide in people with HIV-associated lipohypertrophy: A randomised, double-blind, placebo-controlled phase 2b single-centre clinical trial. Lancet Diabetes Endocrinol..

[97] Thomas T.S., Srinivasa S. (2024). Weighing in: Glucagon-like Peptide-1 Receptor Agonism for Persons with HIV. Top. Antivir. Med..

